# Some Observations on Peripneumony

**Published:** 1814-11

**Authors:** W. Robertson

**Affiliations:** Berwick on Tweed


### *For the Medical and Physical Journal.*

### *Some Observations on Peripneumony; by* W. Robertson, M.D. A.O.L. and C.C.E.

"Ex quo (pulmone scill.) vehemens & acutus morbus oritur, quem περιπνευμονιαν Græci vocant." *Celsus de Med.* p. 224.

INFLAMMATION of the lungs is a disease so rapid in its progress, and at the same time so dangerous, not only from the usual terminations of inflammation, but also from glutinous, bloody, and serous effusions into the cells of the lungs, often producing suffocation, as to require the active exertions of the physician in every stage of the affection.

Boerhaave and his commentator Van Swieten are very solicitous to establish all the minute subdivisions of this disease: the consequence has been, much confusion and repetition; while Hoffman, Cullen, and Gregory have, by a praiseworthy simplification, dissipated the obscurity which formerly enveloped the study of this malady. Two divisions now remain—peripneumony and pleurisy, where the lungs, and where the membranes of the chest, are inflamed: the latter we do not propose to notice here. The first species is characterised by a dull pain in the side or breast, often a sense of oppression,* only accompanied by a cough and dyspnœa.† This affection is frequently the offspring of catarrh, particularly in old people, coming on with a cold stage, and usual symptoms of pyrexia. The pulse is generally soft, and often does not exceed 90 or 100 strokes in a minute.‡ The accompanying anxiety, oppression, and dyspnœa, appear to exist independent of the pain or cough, and have no remission. The cells of the bronchiæ seem

---

* "Id genus morbi plus periculi quam doloris habet." *Celsus*, p. 224.

† Galen, de Locis Affectis, p. 475, observes, "quum vero spirandi difficultati cum angustia & gravitate febris acuta simul accedit, est ille affectus inflammatio pulmonis;" and Celsus, de Med. p. 224, writes thus of peripneumony: "Pulmo totus afficitur. Hunc casum ejus subsequitur tussis, &c. Præcordiorum totiusque pectoris gravitas, spiritus difficultas, magnæ febres, continua vigilia, cibi fastidium, tabes." And Hippocrates describes this disease in few words: "Tussis detinet, orthopnœa, et anhelatio." *De Morbis*, tom. vii. p. 574.

‡ Galen asserts of the pulse, "peripneumonicorum magnus est & undon quid habens, et obscurus, & mollis, similiter ac pulsus lethargicorum." *De Pulsibus.*—It may be here remarked, that acute pain is generally accompanied (as in pleurisy) with a hard and frequent pulse, while the inverse of the position most commonly holds, as in this disease.

contracted and compressed, in consequence of the inflammation, and exhalation takes place into those parts of the lungs which escape this compression, while a spasmodic action affects both the muscles of the larynx and of the chest, as an induction of pain. The dyspnœa sometimes amounts to orthopnœa, but generally the patient lies easiest on the sound side, though this is occasionally reversed, and in some instances the back is the position resorted to.* In severe cases, the patient can breathe only in the half-erect posture, with a slight inclination of the head, thus rendering the action of the diaphragm more free. The cough from the beginning is for the most part accompanied with some expectoration, frequently tinged with blood, and is ejected with tolerable facility. The sputa at an early period are thin and viscid, but, as the malady advances, assume a white thick opaque form, and denote the resolution of the inflammation,† though there are cases on record of resolution having occurred without any expectoration. As the disease proceeds, a considerable intumescence and inflation of the face supervene, from the impeded circulation through the lungs principally, and consequent accumulation in the jugular veins, while there often exists an altered determination of blood to the head, with increased action in its arteries, producing vertigo, intense head-ach, and delirium, which latter sometimes occurs early in the disease. The face often assumes a livid hue, the eye-lids swell, and the lips become black, indicative of venous accumulation and deficient oxygination. It is of importance to observe, that, though the pain is not violent, and often confined to a single spot, yet very extensive and most active inflammation frequently exists under these circumstances, both in the parenchymatous and pleuritic affections. The pain sometimes darts from one side to another, or to the back, demonstrating a severe disease: occasionally it remits, and the young practitioner is apt to mistake the disease, particularly if attended by moderate pyrexia. The patient is supposed to labour under asthma, catarrh, or hydrothorax, till the pain returns, which it often does with redoubled violence, dissipating all discrepance of opinion in regard to the nature of the disease.

* The position of patients was much attended to by Hippocrates, and influenced his prognosis: " Si in morbi vigore ægrotus velit residere hoc in omnibus quidem acutis morbis malum est, pessimum vero est in peripneumoniis." *In Prognosticis,* tom. viii. p. 603.

† " Sputum vero flavum mixtum cum pauco sanguinis in peripneumonicis, in initio morbi, excretum, salutare est & valde confert." *Hippoc. in Prognost.*

The diagnosis and prognosis will be understood from an attentive consideration of the symptoms.

In addition to the terminations of inflammation in general,* this disease has a conclusion peculiar to itself, to wit, an effusion of blood and serum into the cellular texture of the lungs, often producing suffocation. This, indeed, is the ordinary termination when the disease proves fatal, dissection in almost every instance affording ample testimony that such effusion had taken place, and that exudation from the surfaces of the opposed pleuræ had cemented, by its viscid crust, the two membranes. The pericardium also, and, what is usually denominated, cavity of the thorax, evince similar unequivocal demonstrations of high vascular action, under the forms of bloody and serous effusions. These adhesions, doubtless, often take place from slight inflammations, in consequence of juxta-position and similarity of structure of the pleura, but not to the extent usually observed in subjects which have fallen a sacrifice to this disease. Effusion not unfrequently takes place without proving fatal; occasionally, however, this event occurs suddenly, when, from the mildness of the preceding symptoms, it was not expected, and often a few hours only previous to death. Sometimes a gradual and painful suffocation comes on, accompanied by great anguish and delirium, while the patient is totally unable to expectorate the collected sputa from the failure of the vires vitæ. The lungs, after death, in such cases, generally sink in water, and much resemble in colour and density the viscus of the liver, demonstrating the abolition of the air cells, the effects of inflammation and effusion conjointly. Resolution frequently takes place even where no remedies have been used, announced by copious bloody expectoration, or profuse diaphoresis, denoting a relaxation of sputic stricture externally, and (sympathetically) internally even as late as the seventh or eighth day of the disease; but we have no doubt that some remission very generally occurs, in these instances, earlier than this period. Sometimes a diarrhœa, a copious sediment in the urine, an hæmorrhage from the nose or anus, an abscess in the cheek or groin, severally prognosticate safety; but the experienced physician will regard the discharge from the lungs, in the form of thick bloody sputa, easily ejected, as the most certain criterion of approaching convalescence. Hippocrates justly observes,

* We do not propose noticing here the terminations in suppuration, hydrothorax, and gangrene. See Huxham, Burserius, Inst. Med. Pract. vol. iv. part i. For dissections, see Morgagni.

"qui

"qui in peripneumoniis siccis paucæ concocta educunt, metuendi sunt." p. 872.

This disease occurs much more frequently in adults from fifty to seventy years of age, than in others, and generally attacks the male. It is a disease of the strong, vigorous, and plethoric, for the most part. A previous peripneumony strongly predisposes (occurring sometimes periodically), as do asthma, catarrh, and dyspnœa, from whatever cause, also malconformation. The exciting causes are especially cold applied when the body had been previously heated, whether as directed to the surface or lungs; violent exercise in a plethoric state of the system, of the body generally, or of the lungs individually. Intemperance is a frequent predisposing and also exciting cause of peripneumony. The disease is frequently epidemic, and symptomatic of contagious diseases, though not depending on any specific contagion for its own generation.

From the importance of the organ attacked in this disease, the remedies ought to be applied as soon as possible, and in full vigour. Every part of the plan termed antiphlogistic (with the exception of the application of cold)* should be rigorously enjoined, the indications being to resolve inflammation, and to subdue inordinate action in the heart and arteries. To accomplish these purposes, copious and repeated bleedings from a large orifice are to be had recourse to as early in the disease as possible: a few ounces of blood will not suffice, for, as Botallus remarks, "Nunc huic satis fuerit missio sanguinis unciarum decem aut duodecim? non certe, sed librarum vel duarum vel etiam trium."† It is a principal remedy in the first days of the disease, but, if neglected in the beginning, or if the symptoms have not yielded to its influence, and threatening effusion and suffocation, with collapse of the system, ensue, it will not bring safety, it will accelerate the unhappy termination; but local bleeding may be employed with some advantage. After the first bleeding, a large blister ought to be laid on the seat of the pain, and repeated if necessary, while the detraction of blood is to be recurred to in the evening, for little relief is frequently obtained from a first venesection. In the early days of the disease, this remedy is to be had recourse to, till the breathing is relieved, but syncope ought not to be induced in peripneumony, as suffocation might ensue, inde-

---

* See Report of Diseases at the commencement of this Number, under the article "Measles."—Editors.

† De curatione per sanguinis missionem.

pendent of other causes for its prohibition. We ought at the same time to keep in view, that blood-letting in the old, in the intemperate, and in peripneumonia notha, must be kept within certain limits, and that the induction of extreme debility (and its not unfrequent consequence, hydrothorax) is as dangerous as the disease we are combating. The bowels are to be kept soluble by laxative clysters, and if any opening medicine is necessary, it must be of the mildest class. The patient is to be kept as much as may be in the erect posture, and the heat of his chamber ascertained to be under 60° of Fahrenheit. The mucilaginous and demulcent drugs may have a place, and the usual drinks (in a tepid state) be given *ad libitum.* These are the remedies in the early period of peripneumony in the strong and plethoric; but it is to that stage of the disease where secondary symptoms of the most alarming tendency, the effects of inordinate action, arise, that the author is solicitous to call the attention. Where the breathing is heavy, laborious, and oppressed, where the face is bloated and livid, where the pulse is small, hesitating, and irregular,* where the countenance shews unspeakable anxiety, the eyes swollen, the lips full and livid, the nostrils dilated, the head not clear, and a clammy sweat bedews the face and breast only. If any medicine can, under such perilous symptoms, bring healing and safety, it is the rapid introduction of mercury into the system, with a liberal use of diffusible stimuli. Under this regimen, patients who were rapidly hastening to the tombs of their fathers, have been saved. A grain of the submuriate of mercury, conjoined with opium, and exhibited, according to the urgency of the symptoms, every one, two, or three hours, till the gums are affected, by exciting a new train of action, in place of the morbid febrile one about to terminate, very generally brings a speedy alleviation of all the dangerous symptoms. A certain quantity of wine, and preparations thereof, should be enjoined, and the blisters to the head and side (and to the thighs and legs if re-action does not advance) are to be repeated, of ample dimensions. In fact, I consider the circumstances of the disease above described as similar and analogous to those of the secondary stage of the endemic remitting fevers of hot countries, when the depletory means have not been employed in time, or have proved ineffectual in preventing the induction of alarming secondary symptoms. Where the stomach, liver,

* Aretæus observes, "pulsus parvi, frequentissimi & deficientes, quando ipsis mors proxima est." *De Dig. Morb. Acut.* lib. 2, cap. i. p. 11.

or or brain, are threatened with disorganization, from inflammation, injection of blood, and new arrangements; or rather where this process of disintegration has already commenced, and where we have seen the best effects to result from the rapid introduction of mercury, and liberal exhibition of diffusible stimuli; the warm bath should be appended to these means, and is a powerful remedy; but the purging so necessary in endemic fever, is here to be sedulously avoided. Calomel and opium, frequently repeated, with draughts of warm wine (or wine and water), must be liberally administered, till the pulse rises and becomes regular, when the latter must be diminished, or withdrawn altogether. The mercury must be carefully attended to, and its effects on the bowels anticipated and obviated, for purging is a dangerous occurrence at every period after expectoration has commenced.

The practice of exhibiting calomel and opium in some of the genera of febres and phlegmasiæ is not new. Dr. Hamilton, of Lynn Regis, mentions his having done so in the ninth volume of the Edinburgh Medical Commentaries, but principally during the inflammatory stage (premising, however, venesection, &c.) of this and other diseases of the order of phlegmasiæ. To me it appears that mercury produces its most beneficial effects at that period when inordinate febrile action has caused, or is about to produce, an incipient venous paralysis and disorganization in the viscus, the immediate seat of the disease. It is at this time that the mercurial fever or excitement should commence, and if the atony induced by inordinate action has not encroached too far on the powers of life, immediate re-action may be expected, which is to be kept up for a sufficient length of time, till the dangerous collapse of the system is obviated, a return of the bloody sputa induced, a gentle ptyalism engendered, or, in short, till the pulse becomes larger and fuller, and many of the dangerous symptoms disappear.

In addition to the immediate good effects of mercury, I have also to adduce its influence in often preventing the occurrence of the usual sequelæ of inflamed viscera, a long train of chronic misery.

In some future communication, I hope to remit some cases illustrative of the beneficial effects of mercury in the secondary stages of visceral inflammation, and have now only to beg excuse for trespassing so long on your valuable pages.

*Berwick on Tweed,* W. ROBERTSON, M.D.

*June* 28, 1814.

